# Supplementary material for: No Evidence of Temperature-Driven Antimicrobial Resistance in Salmonella Bacteraemia in Queensland, Australia
Source: Antibiotics (Basel). 2025 Dec 16;14(12):1274. doi: 10.3390/antibiotics14121274 (PMC12729408; doi:10.3390/antibiotics14121274)

Supplementary Tables and Figures

Table S1 : Power calculations for the models

Antimicrobial	Events (n)	Power for RR=1.5*	Min Detectable RR (80%)	Assessment
**Gentamicin**	156	82.3%	1.5	Adequate
**Cephalosporins**	152	84.4%	1.5	Adequate
**Ciprofloxacin**	63	67.9%	2.0	Moderate
**Ampicillin**	35	49.3%	2.0	Limited

*RR = Relative Risk per 1°C. Power from 1,000 simulations, α=0.05.

Table S2. Distributed Lag Non-linear model Results for temperature-AMR associations.

Antimicrobial	Temperature Variable	N Events	N Obs	Model	Wald χ^2	df	P-value	Precip β	Precip P
Gentamicin	Mean Temperature	156	117	DLNM	4.592	6	0.597	0	0.709
Gentamicin	Maximum Temperature	156	117	DLNM	1.871	6	0.931	0	0.531
3rd-generation Cephalosporins	Mean Temperature	152	117	DLNM	3.414	6	0.755	0	0.432
3rd-generation Cephalosporins	Maximum Temperature	152	117	DLNM	0.536	6	0.997	0	0.410
Ciprofloxacin	Mean Temperature	63	NA	GAM	NA	NA	NA	NA	NA
Ciprofloxacin	Maximum Temperature	63	NA	GAM	NA	NA	NA	NA	NA
Ampicillin	Mean Temperature	35	NA	GAM	NA	NA	NA	NA	NA

Figure S1. Seasonal variations in Cephalosporin resistance.

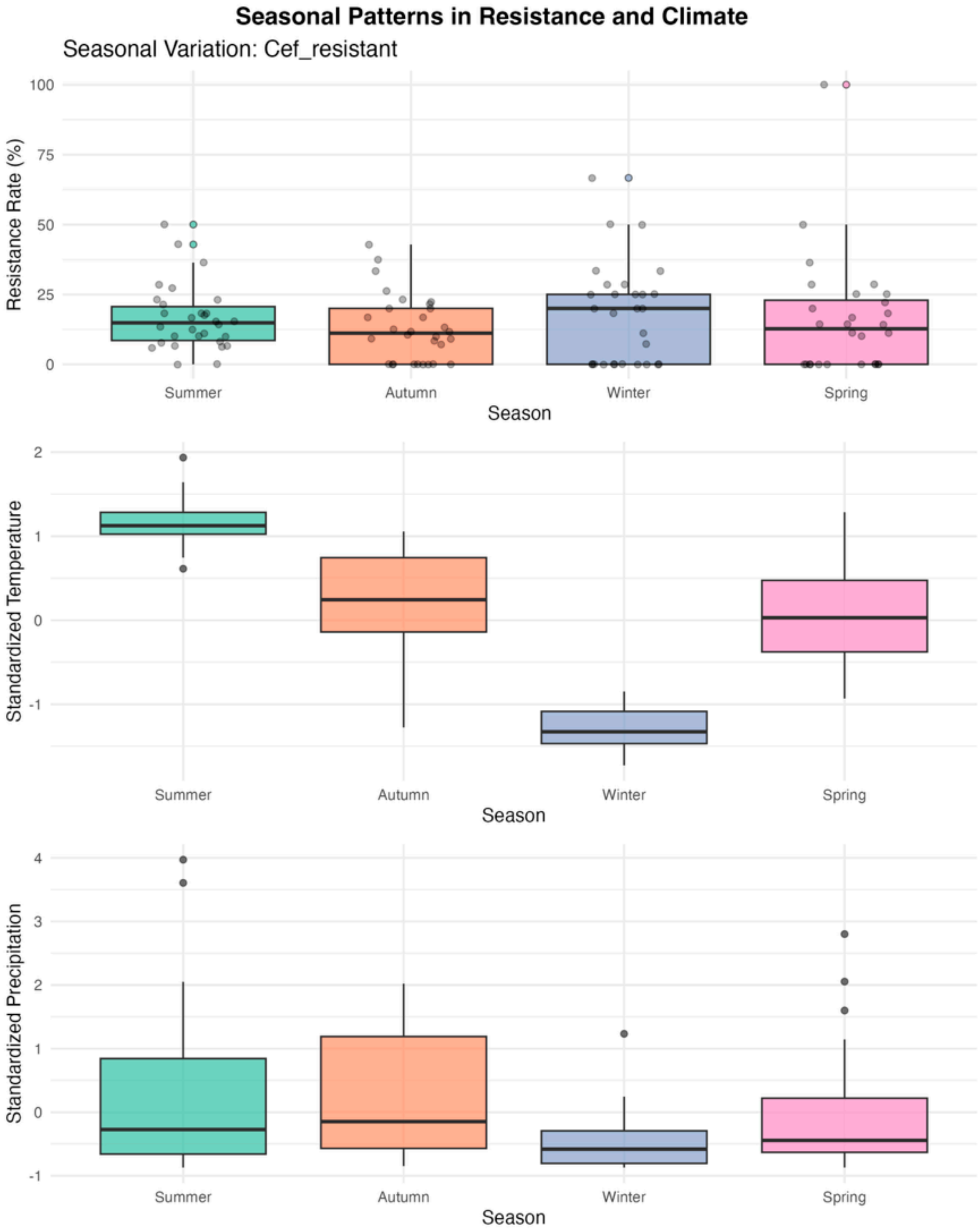

Supplement: Supplementary file 1 [file antibiotics-14-01274-s001.zip › antibiotics-4036296-supplementary.pdf]
